# White matter integrity as a marker for cognitive plasticity in aging

**DOI:** 10.1016/j.neurobiolaging.2016.07.007

**Published:** 2016-11

**Authors:** Ann-Marie Glasø de Lange, Anne Cecilie Sjøli Bråthen, Håkon Grydeland, Claire Sexton, Heidi Johansen-Berg, Jesper L.R. Andersson, Darius A. Rohani, Lars Nyberg, Anders M. Fjell, Kristine B. Walhovd

**Affiliations:** a Research Group for Lifespan Changes in Brain and Cognition, Department of Psychology, University of Oslo, Oslo, Norway; b Department of Physical Medicine and Rehabilitation, Unit of Neuropsychology, Oslo University Hospital, Oslo, Norway; c Oxford Centre for Functional Magnetic Resonance Imaging of the Brain (FMRIB Centre), Nuffield Department of Clinical Neurosciences, University of Oxford, Oxford, UK; d Umeå Center for Functional Brain Imaging, Umeå University, Umeå, Sweden

**Keywords:** Cognitive training, Plasticity, Aging, White matter, Diffusion tensor imaging, Memory

## Abstract

Age-related differences in white matter (WM) integrity are substantial, but it is unknown whether between-subject variability in WM integrity influences the capacity for cognitive improvement. We investigated the effects of memory training related to active and passive control conditions in older adults and tested whether WM integrity at baseline was predictive of training benefits. We hypothesized that (1) memory improvement would be restricted to the training group, (2) widespread areas would show greater mean diffusivity (MD) and lower fractional anisotropy in older adults relative to young adults, and (3) within these areas, variability in WM microstructure in the older group would be predictive of training gains. The results showed that only the group receiving training improved their memory. Significant age differences in MD and fractional anisotropy were found in widespread areas. Within these areas, voxelwise analyses showed a negative relationship between MD and memory improvement in 3 clusters, indicating that WM integrity could serve as a marker for the ability to adapt in response to cognitive challenges in aging.

## 1 Introduction

Brain functions can be modified in response to changing environments and demands throughout the life span (Bavelier et al., 2012, Dahlin et al., 2008, Lovden et al., 2010). However, the potential for cognitive improvements varies substantially among older individuals (Bherer, 2015, West et al., 2002), and we do not know which brain characteristics can account for this. The aim of this study was to directly address this question, by testing whether individual variability in white matter (WM) microstructure at baseline could predict cognitive plasticity, here defined as the extent of behavioral benefit after a memory-training intervention. We hypothesized that individual variability within WM regions vulnerable to age differences would be particularly predictive of cognitive benefit in the older group, and that reduced WM integrity would be associated with lower training gains.

A number of studies indicate that age-related variance in cognitive functioning is related to individual variability in the WM microstructure (Madden et al., 2012). The predominant findings from diffusion tensor imaging studies are increased mean diffusivity (MD) and decreased fractional anisotropy (FA) with aging, albeit accompanied by substantial individual variability (Bennett and Madden, 2014, Salami et al., 2012, Sexton et al., 2014, Westlye et al., 2010). MD represents the mean molecular motion independent of tissue directionality and is suggested to relate to cellular properties such as size and integrity (Basser, 1995, Pierpaoli et al., 1996). Evidence suggests that FA is related to restricted molecular motion caused by directionally oriented microstructures such as myelin sheaths and axonal cell membranes (Beaulieu, 2002, Pierpaoli et al., 1996). WM integrity can be further characterized by axial diffusivity (AD) and radial diffusivity (RD), which represent the rate of diffusion along the primary and secondary axes of the diffusion ellipsoid, respectively (Bennett and Madden, 2014).

Although the exact neurobiological underpinnings of diffusion metrics are unclear, MD and FA are, to some extent, reflective of WM integrity (Zatorre et al., 2012). While we recognize the uncertainties regarding the specific mechanisms underlying diffusion metrics, we will, for simplicity, refer to WM integrity when discussing overall diffusion metrics.

The deterioration in WM microstructure observed in aging has been suggested to follow a posterior to anterior gradient with a greater magnitude of change in frontal regions (Bennett et al., 2010, Burzynska et al., 2010, Davis et al., 2009). The notion that frontal areas are particularly vulnerable to age-related changes has been linked to evidence showing stronger relationships between cognition and WM integrity in frontal regions (Madden et al., 2012). However, the general evidence for relationships between cognition and specific regions of WM is, thus far, unconvincing (Salthouse, 2011), and the regional characteristics of age-related changes in WM microstructure are still unclear (Barrick et al., 2010, Davis et al., 2009, Sexton et al., 2014, Westlye et al., 2010).

Recent years have been marked by a considerable interest in the effects of physical and cognitive training on both cognition and the brain. Studies involving cognitive training paradigms have shown positive training effects in both young and older samples (Burki et al., 2014, Jones et al., 2006, Li et al., 2008, Lovden et al., 2010), where the interindividual variability in training outcome tends to be larger in older groups (Bherer et al., 2006, West et al., 2002). Plotted data often reveal substantial individual variability in cognitive improvement in older adults (Engvig et al., 2012, Hofstetter et al., 2013, Lovden et al., 2012), which emphasize the need to investigate factors underlying the variation in potential for cognitive improvement. The neurological underpinnings of cognitive plasticity are commonly examined in the view of an interactive process between brain and cognition, associating cognitive improvement with change in structural and functional brain measures. Some studies have demonstrated relationships between altered WM microstructure and cognitive improvement after various training programs (Engvig et al., 2012, Hofstetter et al., 2013, Mackey et al., 2012, Nordvik et al., 2012, Schlegel et al., 2012, Scholz et al., 2009). However, individual variation in WM integrity is largely unexplored as a marker for cognitive plasticity.

Here, we aimed to study the effects of a memory-training intervention in older adults relative to active and passive control groups. Training gain was measured using standardized residuals, that is, training effects independent of baseline scores. The training group received 10 weeks of memory training aimed at improving serial verbal recollection memory by implementing the mnemonic technique method of loci (MoL) (Bower, 1970). This method is shown to improve serial recall substantially in older adults (Engvig et al., 2012, Kliegl et al., 1990). The active control group followed an identical schedule as the memory-training group but focused on popular scientific themes as opposed to specific memory training. Areas of significant age differences in MD and FA at baseline were identified in 104 older adults distributed across the different conditions (memory training, passive, and active control) relative to 52 young adults. A conjunction mask based on the areas showing age differences in both MD and FA was used as a region of interest (ROI) for further analyses. We then investigated whether interindividual variation in WM microstructure in areas showing significant age differences was predictive of benefit from cognitive training in the older group. The anchoring of analyses in brain age differences was chosen to investigate markers of cognitive plasticity in aging specifically. It was hypothesized that (1) only the training group would show significant improvement in memory performance after 10 weeks of memory training, (2) widespread areas would show age differences in terms of increased MD and decreased FA with advancing age, and (3) within these areas of age differences, rather than in areas not showing age differences, interindividual variability in WM microstructure in the older group would be predictive of cognitive gains after training, especially in frontal areas hypothesized to show the most prominent age differences.

## 2 Methods and materials

### 2.1 Sample

The sample was drawn from the ongoing project Neurocognitive Plasticity at the Research Group for Lifespan Changes in Brain and Cognition, Department of Psychology, University of Oslo. All procedures were approved by the regional ethical committee of Southern Norway, and written consent was obtained from all participants. Participants were recruited through newspaper and webpage adverts and were screened with a health interview. Participants were required to be either young or older (in or around their 20s or 70s, respectively) healthy adults, right handed, fluent Norwegian speakers, and have normal or corrected to normal vision and hearing. Exclusion criteria were history of injury or disease known to affect central nervous system function, including neurological or psychiatric illness or serious head trauma, being under psychiatric treatment, use of psychoactive drugs known to affect central nervous system functioning, and magnetic resonance imaging (MRI) contraindications. Moreover, for inclusion in the present study, participants were required to score ≥26 on the Mini-Mental State Examination (Folstein et al., 1975) and have scores within normal range (±2 standard deviations [SDs] from mean) for age and sex on the 5-minute delayed recall subtest of the California Verbal Learning Test II (Delis et al., 2000). Three persons in the older group were excluded based on these criteria. All participants further had to achieve an IQ above 85 on the Wechsler Abbreviated Scale of Intelligence (Wechsler, 1999). All scans were evaluated by a neuroradiologist and deemed to be free of significant injuries or conditions. Only participants who completed MRI scanning in addition to 2-assessment sessions were included in the current analyses. Ten of the older participants dropped out after 1 scan, 9 in the training group, 0 in the active control group, and 1 in the passive control group. These participants dropped out either because the particular time frame for assessment was inconvenient for them or because the participation was too time consuming. At the time of the present study, 52 younger and 104 older adults—a total of 156 participants—fulfilled the inclusion criteria. Sample demographics are listed in Table 1. The group of older participants who dropped out performed lower on the California Verbal Learning Test 5-minute recall (mean = 7.67.1, SD = 4.33; independent samples *t* test, t = 2.32, *p* = 0.03) and had lower education compared to the rest of the older sample (mean = 12.22, SD = 3.99, independent samples *t* test, t = 2.79, *p* = 0.006). Lower education and cognitive performance among dropouts is commonly observed in longitudinal studies, resulting in a selection effect toward higher functioning older adults (Salthouse, 2014).

### 2.2 Design and memory training program

The participants were assigned to 1 of 3 groups depending on registration date. Some participants started with 10 weeks of memory training (n = 36), some started with 10 weeks participation in the active control group (n = 19), and some started with 10 weeks as passive controls (n = 49). The passive control group also completed 10 weeks of memory training *after* the initial 10 weeks as passive controls, and was included in a larger training group for statistical analyses including their MRI scan and cognitive performance at time point 3. Thus, some participants were included both as controls (the first 10 weeks) and a part of the training group (the last 10 weeks) in the analyses. All participants were examined using MRI and cognitive testing, with a 10-week interval between each assessment. The training group received 10 weeks of memory training including a single group session each week led by a research fellow. The first group session included a presentation of the project, an introduction of the MoL, and an initial word list task consisting of 15 words. The following weekly group sessions included updating of the strategy and a word list task, which was increased by 5 words each week to ensure a continuous challenge. However, the participants were encouraged to individually adjust the difficulty level to match their capacity, with the aim of achieving a challenging but manageable training level across all the participants. The participants received 8 weekly home assignments, with a minimum requirement that 4 be completed. The home assignments consisted of word lists with various themes and followed the level of difficulty set in the group session the same week. Mean amount of total tasks completed across the participants was 58.2 (74%). The active control group program involved attending popular scientific lectures, in addition to completing home assignments to the same extent as the memory-training group. Mean amount of total tasks completed across participants in the active control group was 55.4 (70%). None of the tasks or lectures in the active control program involved any specific form of memory training. The passive control group received no intervention during the first 10 weeks and then received memory training in the last 10 weeks. Those in the passive control group who completed the training program after the 10 initial weeks were included in a larger training group of 76 participants for the statistical analyses examining the relationship between diffusion tensor imaging measures and change in memory performance.

### 2.3 Image acquisition

A Siemens Skyra 3T MRI scanner with a 24-channel head coil was used (Siemens Medical Solutions; Erlangen, Germany). For the current analysis, a diffusion-weighted echo-planar imaging sequence was applied for each subject (FOV_xy_ = 252 × 256 mm, dimensions = 128 × 130 × 70, voxel size = 1.9626 × 1.9626 mm, slice thickness = 2 mm, repetition time = 9300 ms, and echo time = 87 ms). A total of 64 unique diffusion-weighted volumes were collected at b-value = 1000 s mm^−2^ in addition to 2 non–diffusion-weighted (b-value = 0 s mm^−2^) volumes, 1 acquired with an opposite k-space traversal direction for the purpose of correcting susceptibility artifacts.

### 2.4 Preprocessing

All scan sets were manually checked for gross motion artifacts. The susceptibility-induced field was estimated using the FMRIB Software Library (FSL) tool topup (Andersson et al., 2003) and corrected for along with subject motion and eddy current-induced fields using the eddy tool (Andersson and Sotiropoulos, 2016). Signal dropout caused by subject movement during the diffusion encoding was also detected and corrected (Andersson and Sotiropoulos, 2014). In brief, it compares each acquired slice with a model free prediction and if the observed signal is statistically different (3 SDs) from the prediction, it will be replaced by the latter. The volume was removed if more than 5 slices were replaced, and the entire scan was excluded if more than 6 volumes were removed. An average of 0.34, 0.38, and 0.36 slices per volume across subjects were replaced in the training group, the passive control group, and the active control group, respectively. The number of slices replaced did not differ between the groups [one-way analysis of variance, F (2, 140) = 0.83 *p* = 0.44]. A total of 27 volumes were removed in the training group, 28 and 6 volumes were removed in the passive and the active control group, respectively. No scans were excluded. Nonbrain tissue (skull etc.) was removed using Brain Extraction Tool (Smith, 2002), employing a mask based on the non–diffusion-weighted volume. FA images were created by fitting a tensor model to the preprocessed diffusion data using FMRIB's Diffusion Toolbox (Behrens et al., 2003).

### 2.5 Analysis

All participants' FA data were then processed with the FSL software package Tract-Based Spatial Statistics (TBSS) (Smith et al., 2006). The subjects' FA images were aligned into a common space using FMRIB’s nonlinear image registration tool (FNIRT) (Andersson et al., 2010), which uses a b-spline representation of the registration warp field (Rueckert et al., 1999). Next, the mean FA image was calculated and thinned to create a mean FA skeleton, which represents the centers of all tracts common to the group. The threshold for the mean FA skeleton was set at 0.2, resulting in a mask of 149,473 voxels. Each participant's aligned FA data were then projected onto this skeleton. Nonlinear warps and skeleton projection stages were repeated using MD values.

### 2.6 Test of memory performance

Memory performance was measured using an experimental word list test developed to measure verbal recollection. The test enabled the MoL to be applied, such that the measure of memory performance after the strategy training was closely related to the utilized technique, and thus convenient for measuring training gains. Participants were given 5 minutes to learn as many words as possible in the correct list order, and 10 minutes to recall the words immediately after the learning trial. The learning trial was supervised by a test administrator. To avoid potentialceiling effects, the word list consisted of 100 words. The word lists varied across participants for each testing session. Thus, the words in the list differed between time points. The composition of words in each list was matched on criteria of complexity and how easy and/or difficult they were assumed to transfer to visual imagery. The total number of words recalled from the word list test was used as the score of memory performance before and after the training intervention.

One important issue to consider in training studies is how to measure training outcome or training gain. Common measures include difference scores calculated as performance at time point 2 minus baseline performance (Engvig et al., 2010, Lovden et al., 2010), absolute scores, that is, performance after training (Draganski et al., 2004), and proportional gain such as ratios or percentage scores (Engvig et al., 2014). Absolute difference scores may be suitable as a way of measuring change but do not take into account differences in relative improvement across persons, and ratio scores do not eliminate the influence of baseline variance in analyses (Curran-Everett, 2013). Only residual scores will reflect training effects independent of baseline scores. Here, the baseline variance is removed, thus, the residuals represent the training gain independent of the baseline performance.

### 2.7 Statistical analyses

To examine the effects of the memory-training intervention, a repeated measures analysis of covariance (ANCOVA) was conducted to investigate group × time interaction using age, sex, and baseline memory performance as covariates. Greenhouse-Geisser corrections for violation of sphericity were used. Additional repeated measures ANCOVAs were run for each group, testing the difference in memory performance from baseline to follow up. An independent samples *t* test was performed to compare the number of tasks completed in the training group and the active control group. To investigate the pattern of age differences in tensor-derived values, voxelwise general linear model analyses were carried out on the skeletonized FA and MD values using permutation-based statistics with 10,000 permutations (Nichols and Holmes, 2002) as implemented in *randomise*, part of FSL (Winkler et al., 2014). The significance threshold was set at *p* < 0.05, which is corrected for multiple comparisons across space using threshold-free cluster enhancement (TFCE) (Smith and Nichols, 2009). Sex and motion were used as covariates. Motion was estimated as the mean of the average root mean square displacement value across each diffusion-weighted volume derived from the eddy procedure (Andersson and Sotiropoulos, 2015). In accordance with the hypothesis that variation in WM integrity within areas showing age differences would predict cognitive plasticity, areas showing age differences in both MD and FA were used as an ROI in the analyses of relationships between memory improvement and WM integrity. To test specificity, the analyses were repeated in the areas outside the conjunction mask, that is, the areas *not* showing age effects in MD and FA, and on the full skeleton. To examine whether the magnitude of age differences in MD and FA showed a posterior to anterior gradient, the mean t-scores were calculated across all skeleton voxels for each coronal slice, excluding the slices with <450 voxels (Sexton et al., 2014). A linear regression model using the least squares method was fit to the mean t-scores. To investigate whether the magnitude of the posterior–anterior slopes was related to memory improvement, we correlated the individual slopes in MD and FA with the change in performance on the word list test using age, sex, and motion as covariates. Standardized residuals for each participant were used as the measure of change in memory performance. The residuals were calculated from a linear regression analysis, using memory performance at time point 2 as the dependent variable and memory performance at baseline as the independent variable. To investigate the relationship between memory improvement and MD and FA, we ran general linear models across all voxels in the conjunction mask. To assess effect sizes, mean MD was extracted from significant clusters and correlated with change in memory performance, with age, sex, and motion as covariates.

## 3 Results

### 3.1 Behavioral results

**Fig. 1:**
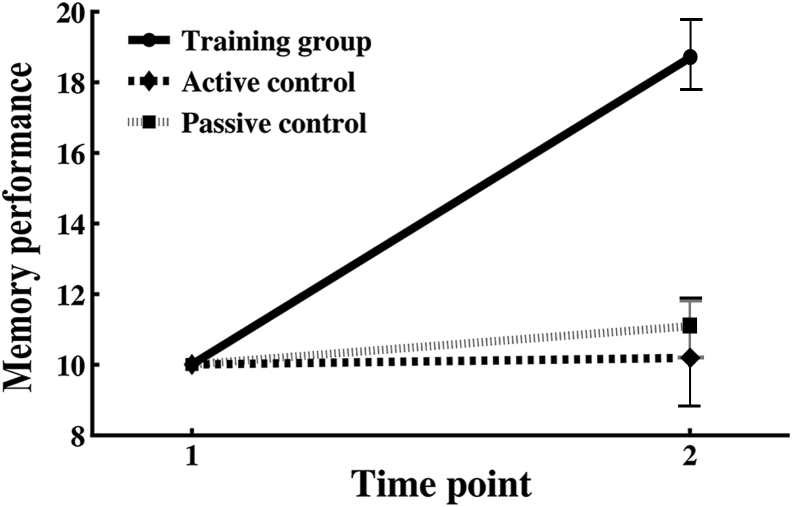
Group differences in memory improvement. Memory scores showed at y-axis. Repeated measures analysis of covariance (Greenhouse-Geisser corrected) with age, sex, and memory performance at baseline as covariates.

The test of group differences in memory performance change revealed a significant group × time interaction [F (2, 14) =23.54, *p* < 0.001], as shown in Fig. 1. Repeated measures analysis of covariance for each group separately showed a significant improvement in memory performance between time points 1 and 2 in the training group [F (1, 72) = 8.00, *p* = 0.006]. No significant change was found in the active control group [F (1, 14) = 0.736, *p* = 0.406] or the passive control group [F (1, 45) = 1.04, *p* = 0.314], respectively. The difference in memory performance did not significantly differ between the 2 control groups (independent samples *t* test, t = 0.833, *p* = 0.408). The 2 control groups were thus treated as a single group for an independent samples *t* test, which showed a significant difference (t = 6.252, *p* < 0.001) in mean change in memory performance between the training group and the merged control group. The number of tasks completed during the training period did not differ between the memory-training group and the active control group (t = −0.68, *p* = 0.49).

### 3.2 Age differences in WM integrity

**Fig. 2:**
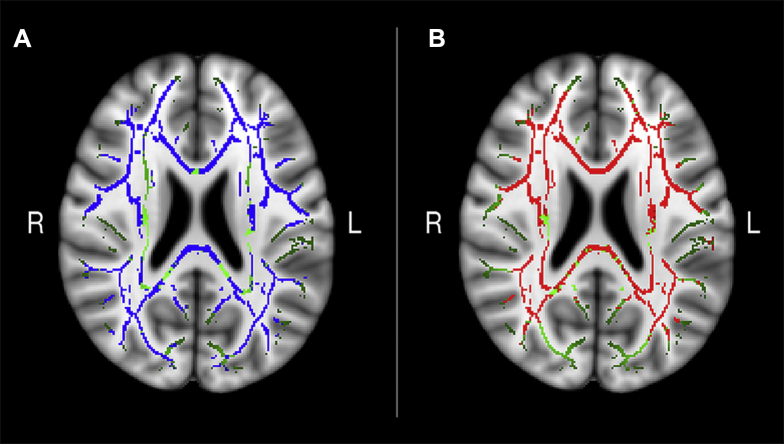
Age differences in WM microstructure. (A) Age differences in FA (blue). (B) Age differences in MD (red). Axial views of Talairach coordinates x = 104, y = 130, and z = 94 overlaid on the mean FA skeleton (green) and the standard MNI152 T_1_ 1-mm^3^ brain template. The results are thresholded at *p* < 0.05 and corrected for multiple comparisons. Abbreviations: WM, white matter; FA, fractional anisotropy; MD, mean diffusivity.

Tests of group differences with sex and motion as covariates showed widespread areas of higher MD (42% of the voxels, *p* < 0.05) and lower FA (65% of the voxels at *p* < 0.05) in older adults, as shown in Fig. 2. No regions showed the opposite pattern (*p* < 0.05). The areas showing age differences in both MD and FA constituted a conjunction mask including 37% of the voxels.

**Fig. 3:**
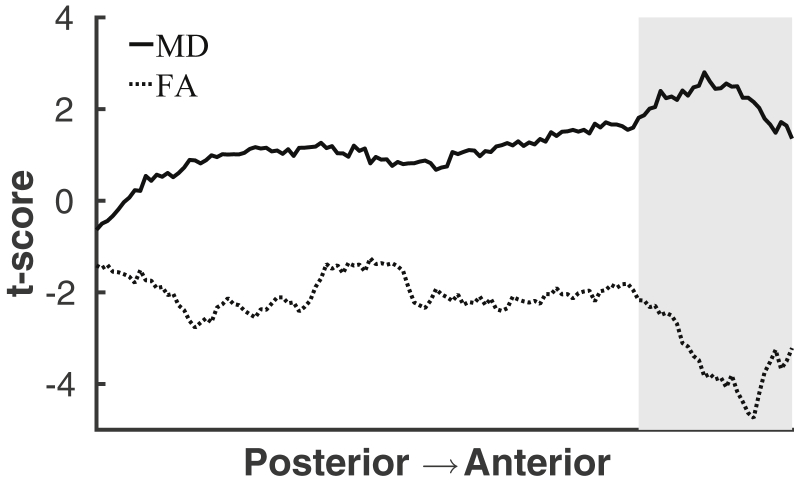
Posterior to anterior gradient of age differences. MD: R^2^ = 0.733, *p* < 0.01, confidence interval [0.0139–0.0172]. FA: R^2^ = 0.385, *p* < 0.01, confidence interval [−0.0155 to −0.0098]. Abbreviations: FA, fractional anisotropy; MD, mean diffusivity.

The mean t-scores for MD and FA in each coronal slice showed increasing magnitude of age effects from posterior to anterior regions, as shown in Fig. 3. Slopes differed from 0 in both MD (R^2^ = 0.733, *p* < 0.01, slope estimate = 0.016) and FA (R^2^ = 0.385, *p* < 0.01, slope estimate = −0.013).

### 3.3 Posterior–anterior slopes and training outcome

**Fig. 9:**
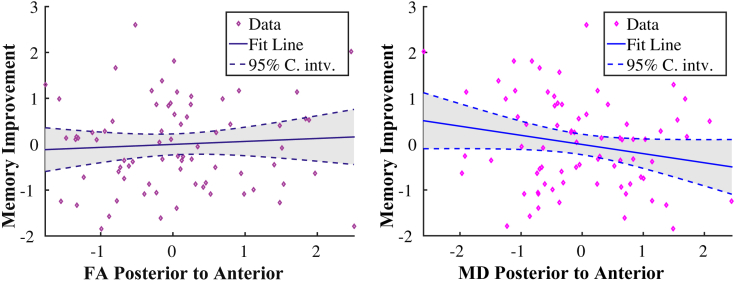
Posterior–anterior slopes and training outcome. Abbreviation: FA, fractional anisotropy; MD, mean diffusivity.

The results showed a weak tendency of a relationship between the magnitude of the posterior–anterior MD slopes and memory improvement (r = −0.196, *p* = 0.097). No relationship was found between the magnitude of the posterior–anterior FA slopes and memory improvement (r = 0.048, *p* = 0.68). Plots are provided in the Supplementary Material (Fig. 9).

Based on the increasing magnitude of age effects from posterior to anterior regions, we investigated the memory improvement in the older participants showing decreased FA in frontal areas and those not showing decreased FA in frontal areas compared to the young subjects. We identified the lowest mean FA value observed in the young sample in a frontal ROI using the anterior slices from slice Y = 143 (standard MNI152 T_1_ 1 mm^3^) and forward, as shown with the gray-shaded area in Fig. 3. The lowest mean FA value in the young sample was used as a cut off to divide the older sample into 2 groups: the individuals showing mean FA values below (N = 52) and above (N = 24), the minimum mean value in the young sample. The results from an independent samples *t* test showed that the group of older adults with frontal FA values above the cut off value exhibited a larger increase in memory performance (mean = 0.35, SD = 1.0) compared to the group with FA values below the cut off (mean = −0.16, SD = 0.95, t = 2.12, *p* = 0.04).

### 3.4 WM integrity and training outcome

**Fig. 4:**
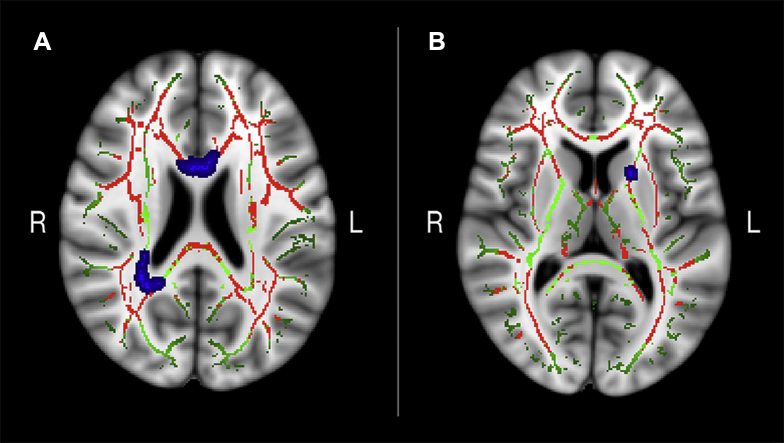
MD clusters related to memory improvement. (A) Cluster 1 (172 voxels) and cluster 2 (53 voxels) shown in blue. Axial view of Talairach coordinates x = 90, y = 137, and z = 94. (B) Cluster 3 (17 voxels) shown in blue, axial view of Talairach coordinates x = 109, y = 136, and z = 82. Clusters are overlaid on the conjunction mask (in red), the mean FA skeleton (in green), and the standard MNI152 T_1_ 1-mm^3^ brain template. The results are thresholded at *p* < 0.05 and corrected for multiple comparisons. Significant areas are dilated for illustrative purposes. Abbreviations: FA, fractional anisotropy; MD, mean diffusivity.

Voxelwise analyses performed within the conjunction mask showed a negative relationship between MD and memory improvement in the older adults in 3 clusters (TFCE-corrected *p* < 0.05; covariates: age, sex, and motion). Effects were located in regions of the anterior corpus callosum (Hofer and Frahm, 2006), the left anterior thalamic radiation, and the right inferior fronto-occipital fasciculus, respectively (Fig. 4). In addition, a positive trend was observed for FA (*p* = 0.065) in anterior parts of the corpus callosum. Results from follow-up analyses on RD and AD revealed a negative relationship between memory improvement and AD in a small cluster of 21 voxels (*p* < 0.05) located in the right inferior fronto-occipital fasciculus, while a negative trend was observed for RD (*p* = 0.053) in anterior parts of the corpus callosum. The results are provided in the Supplementary Material. No relationships were found between MD, FA, RD, or AD and memory improvement outside the conjunction mask based on age differences in FA and MD. A Z test (Steiger, 1980) showed that the correlation with memory inside the conjunction mask was larger than the correlation with memory outside the conjunction mask (Z = −1.734, 1-tailed *p* = 0.04). However, note that this would only show a trend if a 2-tailed test is applied (*p* = 0.08). In the full skeleton, there were trends for negative relationships between memory improvement and MD (*p* = 0.054) and AD (*p* = 0.059) in areas overlapping the right inferior fronto-occipital fasciculus, in addition to a trend for a positive relationship between FA (*p* = 0.06) and memory improvement in the middle and anterior parts of corpus callosum and an area overlapping the premotor cortex area.

To test whether the relationship between WM integrity and memory improvement was specific to training-related gains, we investigated the relationship between WM integrity at baseline and improvement on the Digit Span Backwards test, a test where the participants were asked to render a sequence of numbers backwards. The test showed practice effects in both the control group [repeated measures analysis of variance, F = 30.41(1), *p* = 0.01, mean at baseline = 6.00, SD = 2.12, mean at follow-up = 8.33, SD = 2.12] and in the training group [F = 33.00(1), *p* = 0.01, mean at baseline = 6.22, SD = 2.55, mean at follow-up = 7.43, SD = 2.19]. Thus, the memory training did not seem to influence improvement on this task. No relationship was found between WM integrity at baseline and improvement on the Digit Span Backwards test.

**Fig. 5:**
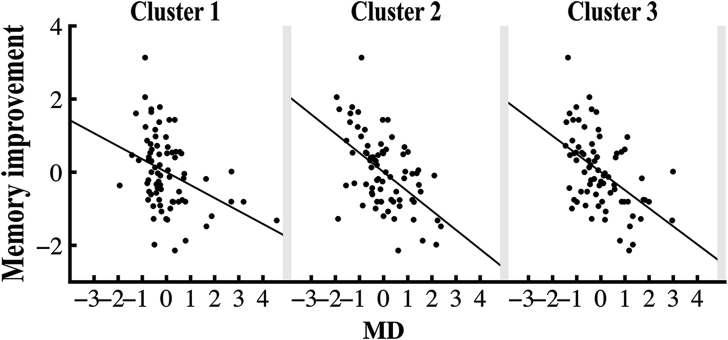
Relationship between memory improvement and MD clusters. MD values in the clusters plotted against memory improvement, corrected for the effect of age, sex, and memory performance at baseline. Linear fit is calculated and shown for each cluster with its respective correlation. Abbreviation: MD, mean diffusivity.

To examine the effect size in the MD clusters, we correlated the mean MD within each cluster separately with change in memory performance, revealing a partial correlation coefficient of r = {−0.36 (*p* = 0.002), −0.53 (*p* = 0.001), −0.49 (*p* = 0.001)} between memory improvement and cluster 1, cluster 2, and cluster 3, respectively, as shown in Fig. 5. To investigate potential outliers among the participants, regression analyses were run with age as the predictor variable and the cluster values in each cluster as criteria variables (Walhovd et al., 2005). Outliers were identified using studentized-deleted residuals, which represent the residual value when the respective case is excluded from the regression. Studentized-deleted residual values exceeding ±3 were treated as outliers. We identified 2 outliers in cluster 1 and 1 outlier in cluster 3. Analyses were rerun without outliers, and the results showed minimal changes as follows: the correlation coefficient decreased from −0.36 to −0.33 (*p* = 0.004) in cluster 1 and increased from −0.49 to −0.52 (*p* = 0.001) in cluster 3.

## 4 Discussion

This study aimed to investigate the effects of a memory-training intervention in older adults relative to active and passive control groups, and to test whether individual differences in WM integrity could serve as a predictor for cognitive gains. Overall, the results showed that (1) only the group receiving the memory-training intervention demonstrated significant improvement in memory performance after 10 weeks. No significant improvement was found in the active and passive control groups. (2) The older adults showed higher MD and lower FA relative to the young adults, with a gradually larger magnitude of age effects from posterior to anterior regions. (3) Within areas of microstructural age differences, regional interindividual MD variability was predictive of cognitive plasticity.

### 4.1 Memory improvement specific to training

In correspondence with previous research, our results show that older individuals can benefit from memory training (Burki et al., 2014, Gross et al., 2014, Jones et al., 2006, Lustig et al., 2009, Nyberg et al., 2003, Rebok et al., 2007). The difference between the training group and the passive and active control groups demonstrated that the memory-training intervention had a unique effect on memory performance measured by the word list test. The inclusion of an active control group strengthens the validity by allowing comparison of effects related to general components of participation and effects related to the specific components of the memory training (Hart et al., 2008, Law et al., 2014). Modest improvement in cognitive function has been found in active control groups (Legault et al., 2011), indicating that increased cognitive activity and social engagement may affect cognitive function in older people (Gallucci et al., 2009). Some, but relatively few, cognitive training studies include active control groups receiving different interventions as a means of comparison (Barnes et al., 2013, Fabre et al., 2002, Legault et al., 2011, Oswald et al., 2006, Schwenk et al., 2010, Suzuki et al., 2012, Zelinski et al., 2011). However, exposure to intervention often varies between conditions (Law et al., 2014), which may compromise the control of factors such as expectations, social contact, and cognitive activity (Hart et al., 2008, Safer and Hugo, 2006, Schwenk et al., 2010). In the current study, the number of tasks, group meetings, and contact with staff were matched between the training group and the active control group, controlling for the possible effect of these factors on memory performance. Furthermore, test sessions and time intervals were held identical for all participants, in order to ensure that test–retest effects would not differ across groups.

### 4.2 Age-related differences in WM integrity

In correspondence with previous cross-sectional research, higher MD and lower FA were found in widespread areas in the older group relative to the young group (Bennett et al., 2010, Burgmans et al., 2010, Burzynska et al., 2010, Charlton et al., 2010, Davis et al., 2009, Kennedy and Raz, 2009, Madden et al., 2009, Marstaller et al., 2015, Pfefferbaum et al., 2000, Voineskos et al., 2012, Westlye et al., 2010). Evidence from longitudinal studies has shown similar patterns of increased MD and decreased FA with advancing age (Barrick et al., 2010, Bender et al., 2015, Engvig et al., 2012, Sexton et al., 2014). Diffusion MRI measurements reflect the restriction of the water molecules, which can be imposed by microstructure such as myelin, microtubules, and cell membranes (Beaulieu, 2002). However, the exact microstructural underpinnings causing a change in the signal cannot be directly inferred, thus, signal changes require cautious interpretation (Wheeler-Kingshott and Cercignani, 2009, Zatorre et al., 2012). The signal may not only be modulated by axon density, membrane permeability, and myelination but also by how axons are laid out within the voxel as the gradient is applied along a given axis (Jones et al., 2013). Although the exact neurobiological underpinnings of diffusion metrics are unclear (Wheeler-Kingshott and Cercignani, 2009, Zatorre et al., 2012), decreased FA and increased MD are suggested to be reflective of microstructural alterations associated with aging (Bennett and Madden, 2014).

The age differences followed a posterior to anterior gradient with increasing magnitude of age differences in anterior regions. Although the posterior to anterior gradient has gained support (Bennett et al., 2010, Burzynska et al., 2010, Davis et al., 2009), recent evidence suggests that this hypothesis may be too simplistic (Sexton et al., 2014, Westlye et al., 2010). However, given our hypothesis that age-associated declines in WM integrity underlie differences in cognitive plasticity, we would expect microstructural differences in the regions showing greater age differences to be particularly predictive of plastic response, which is discussed in the following section.

### 4.3 Variance in WM integrity in older adults is predictive of cognitive plasticity

The magnitude of the posterior–anterior slopes was not significantly related to memory improvement. Although the results showed a tendency that a stronger increase in MD from posterior to anterior regions was negatively related to memory improvement, the results indicate that the individual slopes themselves did not predict change in performance. The relationships between posterior–anterior slopes and training outcome are shown in the Supplementary Material, Fig. 9. The older individuals who showed similar FA values to the young sample in frontal areas showed larger memory improvement than the individuals showing lower mean FA values compared to the young sample. The results from the voxelwise analyses showed a negative relationship between memory improvement and MD within age-vulnerable WM regions, such that lower MD (typically indicative of better WM integrity) was associated with greater performance gains. No relationship was found between WM integrity at baseline and improvement on the Digit Span Backwards test, indicating that MD at baseline was predictive of the training gains related to the memory training rather than commonly observed practice effects due to repeated test exposure (Beglinger et al., 2005). Individual differences in WM integrity in regions of the anterior corpus callosum (Hofer and Frahm, 2006), the left anterior thalamic radiation and the right inferior fronto-occipital fasciculus were particularly predictive of plastic response. The cognitive processes involved in mnemonic strategies are likely to rely on multiple brain areas, and efficient transfer and integration of information between these distributed regions is thus critical. The highlighted clusters in our results may represent regions of importance for information transfer that is beneficial for cognitive improvements after this type of training. This is conjectural, however, as the degree of regional specificity in the relation between cognitive functions and WM integrity is debated (Salthouse, 2011). Although individual studies have shown relationships between cognitive processes and WM integrity in highly specific regions (Kerchner et al., 2012, Zhu et al., 2015), the overall evidence does not currently demonstrate a high degree of regional specificity in the relationship between WM integrity and cognition (Madden et al., 2009, Salthouse, 2011). However, the suggestion that anterior brain areas are particularly vulnerable to age-related decline has gained support (Barrick et al., 2010, Bartzokis, 2004, Brickman et al., 2012, Salat et al., 2005), and some evidence indicates that decline in frontal areas may underlie typical cognitive deficits seen in aging (Bucur et al., 2008, Davis et al., 2009, Rabbitt and Lowe, 2000, Ziegler et al., 2010). Our results indicate that WM integrity, particularly in anterior regions showing prominent age differences, is to some extent predictive of the ability to adapt and benefit from cognitive training. It is, however, unknown whether the effects generalize to other structural features such as gray matter. Thus, investigating whether other brain characteristics can predict potential for cognitive improvement in aging may elucidate the factors underlying individual differences in cognitive plasticity.

## Disclosure statement

The authors have no conflicts of interest to disclose.

## Figures and Tables

**Fig. 6:**
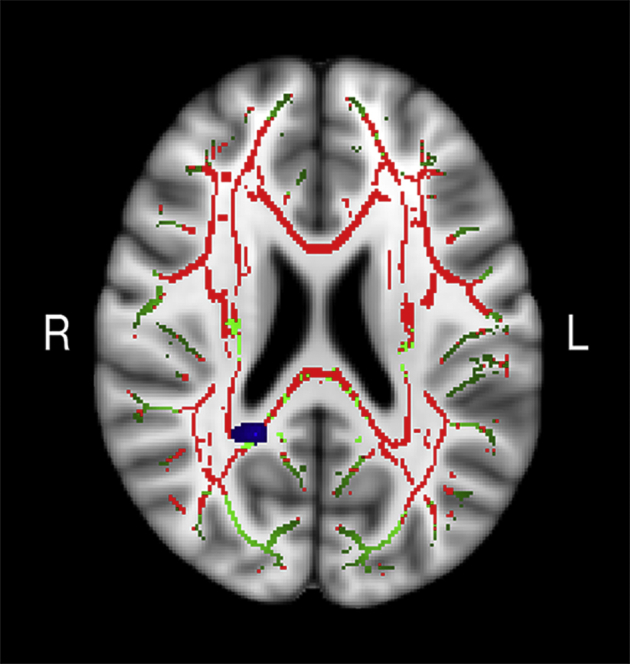
AD cluster related to memory improvement. AD Cluster (21 voxels) shown in blue. Axial view of Talairach coordinates x = 90, y = 137, and z = 94, overlaid on the conjunction mask (in red), the mean FA skeleton (in green) and the standard MNI152 T_1_ 1-mm^3^ brain template. The results are thresholded at *p* < 0.05 and corrected for multiple comparisons. Significant areas are dilated for illustrative purposes. Abbreviations: AD, axial diffusivity; FA, fractional anisotropy.

**Fig. 7:**
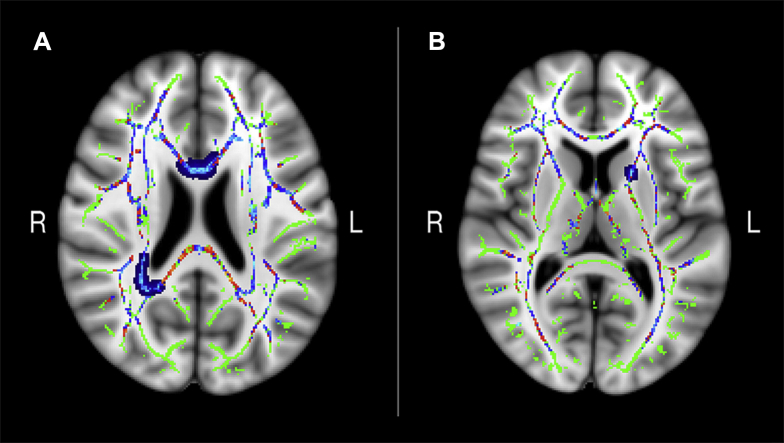
WM integrity and memory performance—general versus specific effects. (A) Cluster 1 and cluster 2 shown in blue. Axial view of Talairach coordinates x = 90, y = 137, and z = 94. (B) Cluster 3 shown in blue, axial view of Talairach coordinates x = 109, y = 136, and z = 82. The clusters are overlaid on the standard MNI152 T_1_ 1-mm^3^ brain template and shown on top of the t-statistics to illustrate general effects below the significance threshold (*p* < 0.05). The blue–lighter blue color map represents areas showing a negative relationship between MD and memory improvement. Lighter blue represents t-values closer to the significant level (*p* < 0.05, t > 2.2). Areas showing a positive relationship between MD and memory improvement is shown in a red-yellow color map where yellow represents t-values closer to the significant level (*p* < 0.05, t > 2.2). Abbreviations: MD, mean diffusivity; WM, white matter.

**Fig. 8:**
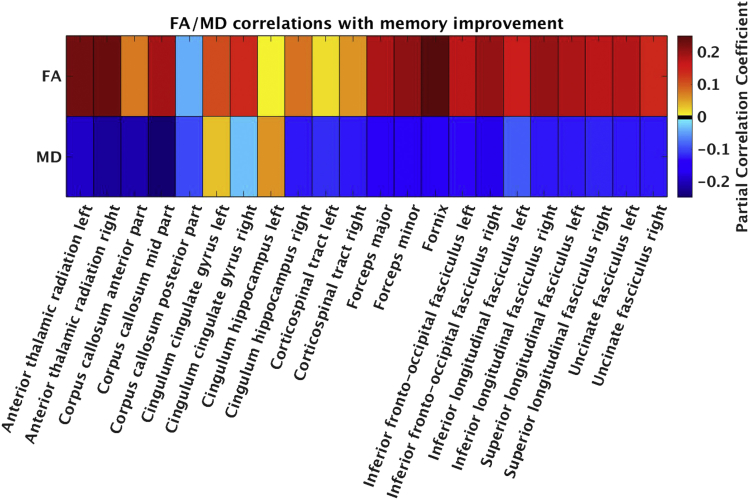
WM integrity and memory performance—individual tracts. Abbreviation: FA, fractional anisotropy; MD, mean diffusivity; WM, white matter.

**Table 1:** Sample demographics

	Full sample				Older adults		
	Young adults (41 females/11 males)		Older adults (67 females/37 males)		Training group (51 females/25 males)	Active control (11 females/7 males)	Passive control (33 females/16 males)
	M ± SD	Range	M ± SD	Range	M ± SD	M ± SD	M ± SD
Age	26.4 ± 3.0	20:31	73.4 ± 2.9	68:82	73.6 ± 3.0	73.5 ± 2.9	73.4 ± 3.2
Education	15.7 ± 1.8	12:18	15.0 ± 2.8	9:21	15.0 ± 2.7	16.2 ± 2.7	14.2 ± 2.6
MMS	29.3 ± 0.9	26:30	28.7 ± 1.3	26:30	28.8 ± 1.2	28.2 ± 1.5	28.8 ± 1.1
IQ	111.9 ± 8.4	88:130	119.8 ± 10.4	88:144	120.3 ± 10.2	121.3 ± 5.6	117.8 ± 11.1
CVLT L	62.0 ± 7.9	41:77	47.7 ± 10.5	21:71	49.1 ± 10.8	50.0 ± 10.0	47.0 ± 10.3
CVLT R	14.1 ± 2.1	8:16	10.3 ± 3.1	4:16	10.5 ± 3.4	11.4 ± 3.3	9.9 ± 2.8

One-way analysis of variance showed significant (*p* < 0.01) differences across age groups in IQ, Mini Mental Status (MMS), CVLT learning (L) and CVLT 5-minutes recall (R) but no differences (*p* = 0.150) in years of education, which includes years of completed grades on each level, for example, primary school, secondary school, high school, master degree, and PhD. One-way analysis of variance showed significant (*p* < 0.03) differences between the active and the passive control groups in years of education (multiple comparisons, Bonferroni). No differences were found between the groups on age, MMS, IQ, CVLT learning, or CVLT 5-minutes recall.

Key: CVLT, California Verbal Learning Test; M, mean; SD, standard deviation.

## References

[bib1] Andersson J.L.R., Jenkinson M., Smith S. (2010). Non-Linear Registration, Aka Spatial Normalisation.

[bib2] Andersson J.L.R., Skare S., Ashburner J. (2003). How to correct susceptibility distortions in spin-echo echo-planar images: application to diffusion tensor imaging. Neuroimage.

[bib3] Andersson J.L.R., Sotiropoulos S.N. (2016). A Gaussian Process Based Method for Detecting and Correcting Dropout in Diffusion Imaging.

[bib4] Andersson J.L., Sotiropoulos S.N. (2016). An integrated approach to correction for off-resonance effects and subject movement in diffusion MR imaging. Neuroimage.

[bib5] Barnes D.E., Santos-Modesitt W., Poelke G., Kramer A.F., Castro C., Middleton L.E., Yaffe K. (2013). The Mental Activity and Exercise (MAX) trial: a randomized controlled trial to enhance cognitive function in older adults. JAMA Intern. Med..

[bib6] Barrick T.R., Charlton R.A., Clark C.A., Markus H.S. (2010). White matter structural decline in normal ageing: a prospective longitudinal study using tract-based spatial statistics. NeuroImage.

[bib7] Bartzokis G. (2004). Age-related myelin breakdown: a developmental model of cognitive decline and Alzheimer's disease. Neurobiol. Aging.

[bib8] Basser P.J. (1995). Inferring microstructural features and the physiological state of tissues from diffusion-weighted images. NMR Biomed..

[bib9] Bavelier D., Green C.S., Pouget A., Schrater P. (2012). Brain plasticity through the life span: learning to learn and action video games. Annu. Rev. Neurosci..

[bib10] Beaulieu C. (2002). The basis of anisotropic water diffusion in the nervous system - a technical review. NMR Biomed..

[bib11] Beglinger L.J., Gaydos B., Tangphao-Daniels O., Duff K., Kareken D.A., Crawford J., Fastenau P.S., Siemers E.R. (2005). Practice effects and the use of alternate forms in serial neuropsychological testing. Arch. Clin. Neuropsychol..

[bib12] Behrens T.E., Woolrich M.W., Jenkinson M., Johansen-Berg H., Nunes R.G., Clare S., Matthews P.M., Brady J.M., Smith S.M. (2003). Characterization and propagation of uncertainty in diffusion-weighted MR imaging. Magn. Reson. Med..

[bib13] Bender A.R., Volkle M.C., Raz N. (2015). Differential aging of cerebral white matter in middle-aged and older adults: a seven-year follow-up. Neuroimage.

[bib14] Bennett I.J., Madden D.J. (2014). Disconnected aging: cerebral white matter integrity and age-related differences in cognition. Neuroscience.

[bib15] Bennett I.J., Madden D.J., Vaidya C.J., Howard D.V., Howard J.H. (2010). Age-related differences in multiple measures of white matter integrity: a diffusion tensor imaging study of healthy aging. Hum. Brain Mapp..

[bib16] Bherer L. (2015). Cognitive plasticity in older adults: effects of cognitive training and physical exercise. Ann. N. Y. Acad. Sci..

[bib17] Bherer L., Kramer A.F., Peterson M.S., Colcombe S., Erickson K., Becic E. (2006). Testing the limits of cognitive plasticity in older adults: application to attentional control. Acta Psychol..

[bib18] Bower G.H. (1970). Analysis of a mnemonic device. Am. Sci..

[bib19] Brickman A.M., Meier I.B., Korgaonkar M.S., Provenzano F.A., Grieve S.M., Siedlecki K.L., Wasserman B.T., Williams L.M., Zimmerman M.E. (2012). Testing the white matter retrogenesis hypothesis of cognitive aging. Neurobiol. Aging.

[bib20] Bucur B., Madden D.J., Spaniol J., Provenzale J.M., Cabeza R., White L.E., Huettel S.A. (2008). Age-related slowing of memory retrieval: contributions of perceptual speed and cerebral white matter integrity. Neurobiol. Aging.

[bib21] Burgmans S., van Boxtel M.P., Gronenschild E.H., Vuurman E.F., Hofman P., Uylings H.B., Jolles J., Raz N. (2010). Multiple indicators of age-related differences in cerebral white matter and the modifying effects of hypertension. Neuroimage.

[bib22] Burki C.N., Ludwig C., Chicherio C., de Ribaupierre A. (2014). Individual differences in cognitive plasticity: an investigation of training curves in younger and older adults. Psychol. Res..

[bib23] Burzynska A.Z., Preuschhof C., Backman L., Nyberg L., Li S.C., Lindenberger U., Heekeren H.R. (2010). Age-related differences in white matter microstructure: region-specific patterns of diffusivity. Neuroimage.

[bib24] Charlton R.A., Barrick T.R., Markus H.S., Morris R.G. (2010). The relationship between episodic long-term memory and white matter integrity in normal aging. Neuropsychologia.

[bib25] Curran-Everett D. (2013). Explorations in statistics: the analysis of ratios and normalized data. Adv. Physiol. Educ..

[bib26] Dahlin E., Nyberg L., Backman L., Neely A.S. (2008). Plasticity of executive functioning in young and older adults: immediate training gains, transfer, and long-term maintenance. Psychol. Aging.

[bib27] Davis S.W., Dennis N.A., Buchler N.G., White L.E., Madden D.J., Cabeza R. (2009). Assessing the effects of age on long white matter tracts using diffusion tensor tractography. Neuroimage.

[bib28] Delis D.C., Kramer J.H., Kaplan E., Ober B.A. (2000). California Verbal Learning Test - Second Edition (CVLT - II).

[bib29] Draganski B., Gaser C., Busch V., Schuierer G., Bogdahn U., May A. (2004). Neuroplasticity: changes in grey matter induced by training. Nature.

[bib30] Engvig A., Fjell A.M., Westlye L.T., Moberget T., Sundseth O., Larsen V.A., Walhovd K.B. (2010). Effects of memory training on cortical thickness in the elderly. Neuroimage.

[bib31] Engvig A., Fjell A.M., Westlye L.T., Moberget T., Sundseth O., Larsen V.A., Walhovd K.B. (2012). Memory training impacts short-term changes in aging white matter: a longitudinal diffusion tensor imaging study. Hum. Brain Mapp..

[bib32] Engvig A., Fjell A.M., Westlye L.T., Skaane N.V., Dale A.M., Holland D., Due-Tonnessen P., Sundseth O., Walhovd K.B. (2014). Effects of cognitive training on gray matter volumes in memory clinic patients with subjective memory impairment. J. Alzheimer's Dis. : JAD.

[bib33] Fabre C., Chamari K., Mucci P., Masse-Biron J., Prefaut C. (2002). Improvement of cognitive function by mental and/or individualized aerobic training in healthy elderly subjects. Int. J. Sports Med..

[bib34] Folstein M.F., Folstein S.E., McHugh P.R. (1975). “Mini-mental state”. A practical method for grading the cognitive state of patients for the clinician. J. Psychiatr. Res..

[bib35] Gallucci M., Antuono P., Ongaro F., Forloni P.L., Albani D., Amici G.P., Regini C. (2009). Physical activity, socialization and reading in the elderly over the age of seventy: what is the relation with cognitive decline? Evidence from “The Treviso Longeva (TRELONG) study”. Arch. Gerontol. Geriatr..

[bib36] Gross A.L., Brandt J., Bandeen-Roche K., Carlson M.C., Stuart E.A., Marsiske M., Rebok G.W. (2014). Do older adults use the method of loci? Results from the ACTIVE study. Exp. Aging Res..

[bib37] Hart T., Fann J.R., Novack T.A. (2008). The dilemma of the control condition in experience-based cognitive and behavioural treatment research. Neuropsychological Rehabil..

[bib38] Hofer S., Frahm J. (2006). Topography of the human corpus callosum revisited–comprehensive fiber tractography using diffusion tensor magnetic resonance imaging. Neuroimage.

[bib39] Hofstetter S., Tavor I., Tzur Moryosef S., Assaf Y. (2013). Short-term learning induces white matter plasticity in the fornix. J. Neurosci..

[bib40] Jones D.K., Knosche T.R., Turner R. (2013). White matter integrity, fiber count, and other fallacies: the do's and don'ts of diffusion MRI. Neuroimage.

[bib41] Jones S., Nyberg L., Sandblom J., Stigsdotter Neely A., Ingvar M., Magnus Petersson K., Backman L. (2006). Cognitive and neural plasticity in aging: general and task-specific limitations. Neurosci. Biobehav. Rev..

[bib42] Kennedy K.M., Raz N. (2009). Pattern of normal age-related regional differences in white matter microstructure is modified by vascular risk. Brain Res..

[bib43] Kerchner G.A., Racine C.A., Hale S., Wilheim R., Laluz V., Miller B.L., Kramer J.H. (2012). Cognitive processing speed in older adults: relationship with white matter integrity. PLoS One.

[bib44] Kliegl R., Smith J., Baltes P.B. (1990). On the locus and process of magnification of age-differences during mnemonic training. Dev. Psychol..

[bib45] Law L.L., Barnett F., Yau M.K., Gray M.A. (2014). Effects of combined cognitive and exercise interventions on cognition in older adults with and without cognitive impairment: a systematic review. Ageing Res. Rev..

[bib46] Legault C., Jennings J.M., Katula J.A., Dagenbach D., Gaussoin S.A., Sink K.M., Rapp S.R., Rejeski W.J., Shumaker S.A., Espeland M.A., Group, S.-P.S (2011). Designing clinical trials for assessing the effects of cognitive training and physical activity interventions on cognitive outcomes: the Seniors Health and Activity Research Program Pilot (SHARP-P) study, a randomized controlled trial. BMC Geriatr..

[bib47] Li S.C., Schmiedek F., Huxhold O., Rocke C., Smith J., Lindenberger U. (2008). Working memory plasticity in old age: practice gain, transfer, and maintenance. Psychol. Aging.

[bib48] Lovden M., Bodammer N.C., Kuhn S., Kaufmann J., Schutze H., Tempelmann C., Heinze H.J., Duzel E., Schmiedek F., Lindenberger U. (2010). Experience-dependent plasticity of white-matter microstructure extends into old age. Neuropsychologia.

[bib49] Lovden M., Brehmer Y., Li S.C., Lindenberger U. (2012). Training-induced compensation versus magnification of individual differences in memory performance. Front. Hum. Neurosci..

[bib50] Lustig C., Shah P., Seidler R., Reuter-Lorenz P.A. (2009). Aging, training, and the brain: a review and future directions. Neuropsychol. Rev..

[bib51] Mackey A.P., Whitaker K.J., Bunge S.A. (2012). Experience-dependent plasticity in white matter microstructure: reasoning training alters structural connectivity. Front Neuroanat..

[bib52] Madden D.J., Bennett I.J., Burzynska A., Potter G.G., Chen N.K., Song A.W. (2012). Diffusion tensor imaging of cerebral white matter integrity in cognitive aging. Biochim. Biophys. Acta.

[bib53] Madden D.J., Bennett I.J., Song A.W. (2009). Cerebral white matter integrity and cognitive aging: contributions from diffusion tensor imaging. Neuropsychol. Rev..

[bib54] Marstaller L., Williams M., Rich A., Savage G., Burianova H. (2015). Aging and large-scale functional networks: white matter integrity, gray matter volume, and functional connectivity in the resting state. Neuroscience.

[bib55] Nichols T.E., Holmes A.P. (2002). Nonparametric permutation tests for functional neuroimaging: a primer with examples. Hum. Brain Mapp..

[bib56] Nordvik J.E., Schanke A.K., Walhovd K., Fjell A., Grydeland H., Landro N.I. (2012). Exploring the relationship between white matter microstructure and working memory functioning following stroke: a single case study of computerized cognitive training. Neurocase.

[bib57] Nyberg L., Sandblom J., Jones S., Neely A.S., Petersson K.M., Ingvar M., Backman L. (2003). Neural correlates of training-related memory improvement in adulthood and aging. Proc. Natl. Acad. Sci. U. S. A..

[bib58] Oswald W.D., Gunzelmann T., Rupprecht R., Hagen B. (2006). Differential effects of single versus combined cognitive and physical training with older adults: the SimA study in a 5-year perspective. Eur. J. Ageing.

[bib59] Pfefferbaum A., Sullivan E.V., Hedehus M., Lim K.O., Adalsteinsson E., Moseley M. (2000). Age-related decline in brain white matter anisotropy measured with spatially corrected echo-planar diffusion tensor imaging. Magn. Reson. Med..

[bib60] Pierpaoli C., Jezzard P., Basser P.J., Barnett A., Di Chiro G. (1996). Diffusion tensor MR imaging of the human brain. Radiology.

[bib61] Rabbitt P., Lowe C. (2000). Patterns of cognitive ageing. Psychol. Res..

[bib62] Rebok G.W., Carlson M.C., Langbaum J.B. (2007). Training and maintaining memory abilities in healthy older adults: traditional and novel approaches. J. Gerontol. Ser. B. Psychol. Sci. Soc. Sci..

[bib87] Rueckert D., Sonoda L.I., Hayes C., Hill D.L.G., Leach M.O., Hawkes D.J. (1999). Nonrigid registration using free-form deformations: Application to breast MR images. IEEE Trans Med Imaging.

[bib63] Safer D.L., Hugo E.M. (2006). Designing a control for a behavioral group therapy. Behav. Ther..

[bib64] Salami A., Eriksson J., Nilsson L.G., Nyberg L. (2012). Age-related white matter microstructural differences partly mediate age-related decline in processing speed but not cognition. Biochim. Biophys. Acta.

[bib65] Salat D.H., Tuch D.S., Greve D.N., van der Kouwe A.J., Hevelone N.D., Zaleta A.K., Rosen B.R., Fischl B., Corkin S., Rosas H.D., Dale A.M. (2005). Age-related alterations in white matter microstructure measured by diffusion tensor imaging. Neurobiol. Aging.

[bib66] Salthouse T.A. (2011). Neuroanatomical substrates of age-related cognitive decline. Psychol. Bull..

[bib67] Salthouse T.A. (2014). Selectivity of attrition in longitudinal studies of cognitive functioning. J. Gerontol. Ser. B. Psychol. Sci. Soc. Sci..

[bib68] Schlegel A.A., Rudelson J.J., Tse P.U. (2012). White matter structure changes as adults learn a second language. J. Cogn. Neurosci..

[bib69] Scholz J., Klein M.C., Behrens T.E., Johansen-Berg H. (2009). Training induces changes in white-matter architecture. Nat. Neurosci..

[bib70] Schwenk M., Zieschang T., Oster P., Hauer K. (2010). Dual-task performances can be improved in patients with dementia: a randomized controlled trial. Neurology.

[bib71] Sexton C.E., Walhovd K.B., Storsve A.B., Tamnes C.K., Westlye L.T., Johansen-Berg H., Fjell A.M. (2014). Accelerated changes in white matter microstructure during aging: a longitudinal diffusion tensor imaging study. J. Neurosci. : official J. Soc. Neurosci..

[bib72] Smith S.M. (2002). Fast robust automated brain extraction. Hum. Brain Mapp..

[bib88] Smith S.M., Jenkinson M., Johansen-Berg H., Rueckert D., Nichols T.E., Mackay C.E., Watkins K.E., Ciccarelli O., Cader M.Z., Matthews P.M., Behrens T.E. (2006). Tract-based spatial statistics: voxelwise analysis of multi-subject diffusion data. NeuroImage.

[bib73] Smith S.M., Nichols T.E. (2009). Threshold-free cluster enhancement: addressing problems of smoothing, threshold dependence and localisation in cluster inference. Neuroimage.

[bib74] Steiger J.H. (1980). Tests for comparing elements of a correlation matrix. Psychol. Bull..

[bib75] Suzuki T., Shimada H., Makizako H., Doi T., Yoshida D., Tsutsumimoto K., Anan Y., Uemura K., Lee S., Park H. (2012). Effects of multicomponent exercise on cognitive function in older adults with amnestic mild cognitive impairment: a randomized controlled trial. BMC Neurol..

[bib76] Voineskos A.N., Rajji T.K., Lobaugh N.J., Miranda D., Shenton M.E., Kennedy J.L., Pollock B.G., Mulsant B.H. (2012). Age-related decline in white matter tract integrity and cognitive performance: a DTI tractography and structural equation modeling study. Neurobiol. Aging.

[bib77] Walhovd K.B., Fjell A.M., Reinvang I., Lundervold A., Dale A.M., Eilertsen D.E., Quinn B.T., Salat D., Makris N., Fischl B. (2005). Effects of age on volumes of cortex, white matter and subcortical structures. Neurobiol. Aging.

[bib78] Wechsler D. (1999). Wechsler Abbreviated Scale of Intelligence.

[bib79] West R., Murphy K.J., Armilio M.L., Craik F.I., Stuss D.T. (2002). Lapses of intention and performance variability reveal age-related increases in fluctuations of executive control. Brain Cogn..

[bib80] Westlye L.T., Walhovd K.B., Dale A.M., Bjornerud A., Due-Tonnessen P., Engvig A., Grydeland H., Tamnes C.K., Ostby Y., Fjell A.M. (2010). Life-span changes of the human brain white matter: diffusion tensor imaging (DTI) and volumetry. Cereb. Cortex.

[bib81] Wheeler-Kingshott C.A.M., Cercignani M. (2009). About “axial” and “radial” diffusivities. Magn. Reson. Med..

[bib82] Winkler A.M., Ridgway G.R., Webster M.A., Smith S.M., Nichols T.E. (2014). Permutation inference for the general linear model. Neuroimage.

[bib83] Zatorre R.J., Fields R.D., Johansen-Berg H. (2012). Plasticity in gray and white: neuroimaging changes in brain structure during learning. Nat. Neurosci..

[bib84] Zelinski E.M., Spina L.M., Yaffe K., Ruff R., Kennison R.F., Mahncke H.W., Smith G.E. (2011). Improvement in memory with plasticity-based adaptive cognitive training: results of the 3-month follow-up. J. Am. Geriatr. Soc..

[bib85] Zhu Z., Johnson N.F., Kim C., Gold B.T. (2015). Reduced frontal cortex efficiency is associated with lower white matter integrity in aging. Cereb. Cortex.

[bib86] Ziegler D.A., Piguet O., Salat D.H., Prince K., Connally E., Corkin S. (2010). Cognition in healthy aging is related to regional white matter integrity, but not cortical thickness. Neurobiol. Aging.

