# Supplementary material for: White matter integrity as a marker for cognitive plasticity in aging
Source: Neurobiol Aging. 2016 Nov;47:74–82. doi: 10.1016/j.neurobiolaging.2016.07.007 (PMC5404118; doi:10.1016/j.neurobiolaging.2016.07.007)
Supplement: Supplementary Materials [file mmc1.pdf]

Supplementary material

WM integrity and training outcome – Axial and radial diffusivity

Analyses stages were repeated on the diffusion metrics Axonal diffusivity (AD) and Radial diffusivity (RD) using the ROI based on age differences in MD and FA. The results revealed a negative relationship between AD and memory improvement in a small cluster of 21 voxels ($p < 0.05$) located in the right inferior fronto-occipital fasciculus as shown in Figure 6, while a trend was observed for RD ($p = 0.053$) in anterior parts of the corpus callosum.

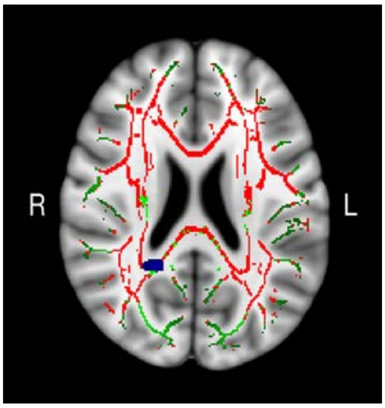

Figure 6. AD cluster related to memory improvement. AD Cluster (21 voxels) shown in blue. Axial view of Talairach coordinates x = 90, y = 137, z = 94, overlaid on the conjunction mask (in red), the mean FA skeleton (in green) and the standard MNI152 T_1 1 mm^3 brain template. The results are thresholded at $p < 0.05$ and corrected for multiple comparisons. Significant areas are dilated for illustrative purposes.

WM integrity and baseline memory performance

To investigate the relationship between baseline performance on the word list test and WM integrity, we ran GLMs across all voxels in the conjunction mask. The results showed no relationships between diffusion metrics and memory performance at baseline.

Baseline memory performance and memory change

To investigate the relationship between memory performance at baseline and memory improvement in the training group, we performed correlations between baseline scores and difference scores (time point 2 minus baseline) on the word list test. No significant correlation was found between baseline performance and improvement.

WM integrity and memory performance – general versus specific effects

The clusters where significant relationships were found between MD and change in memory performance is shown on top of the t statistics in Figure 7. In addition, we investigated the association between WM integrity and memory improvement in a set of extracted WM tracts. The results are illustrated in Figure 8.”

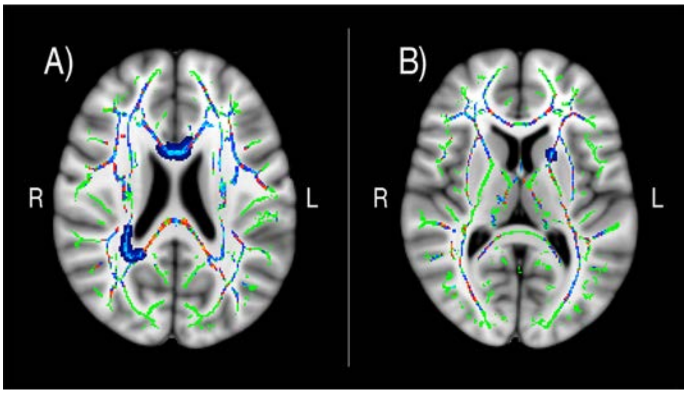

Figure 7. WM integrity and memory performance – general versus specific effects. (a) Cluster 1 and cluster 2 shown in blue. Axial view of Talairach coordinates x = 90, y = 137, z = 94. b) Cluster 3 shown in blue, axial view of Talairach coordinates x = 109, y = 136, z = 82. The clusters are overlaid on the standard MNI152 T_1 1 mm^3 brain template and shown on top of the t-statistics to illustrate general effects below the significance threshold ($p < 0.05$). The blue-lighter blue colour map represents areas showing a negative relationship between MD and memory improvement. Lighter blue represents t-values closer to the significant level ($p < 0.05$, $t > 2.2$). Areas showing a positive relationship between MD and memory improvement is shown in a red-yellow colour map where yellow represents t-values closer to the significant level ($p < 0.05$, $t > 2.2$).

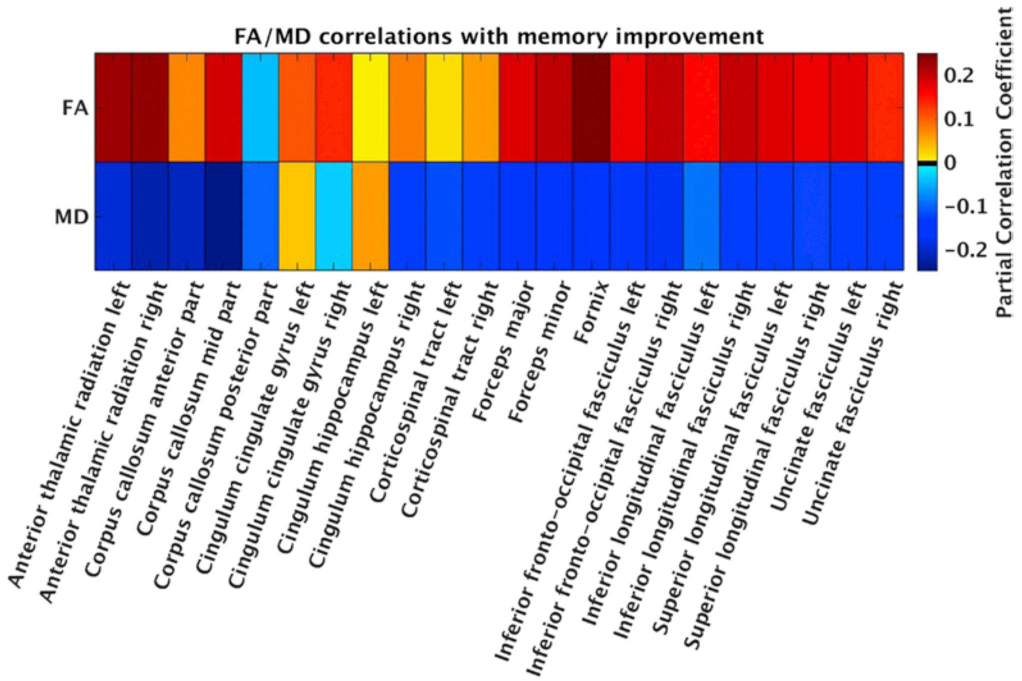

Figure 8.WM integrity and memory performance – Individual tracts.

The results of the analyses of relationships between posterior-anterior slopes and training outcome are shown in figure 8 and 9.

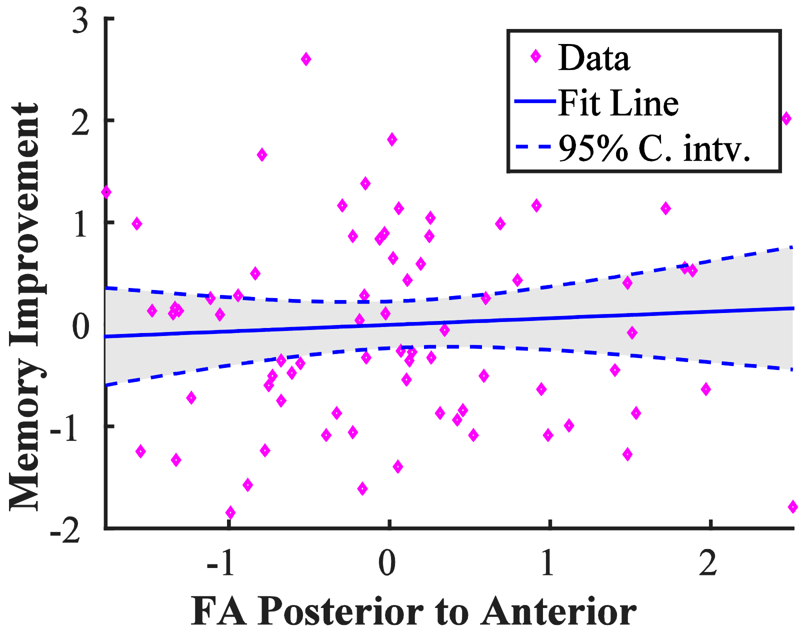

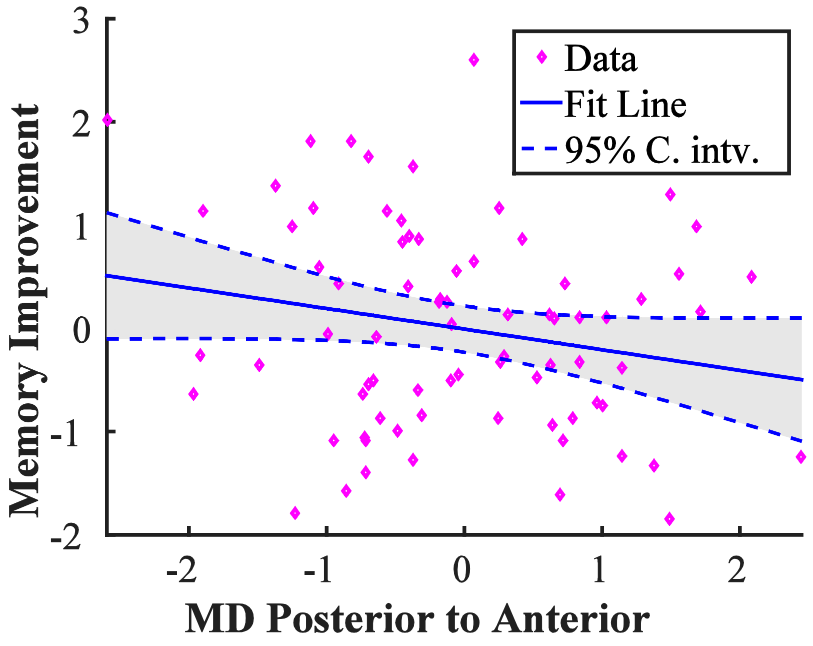

Figures 9 and 10. Posterior-anterior slopes and training outcome.

WM integrity and training gains using difference scores

To investigate whether results varied when using difference scores (time point two minus baseline) as a measure of training gains, we ran voxel wise analyses in the conjunction mask. No relationship was found between difference scores and MD.
